# Nocebo effects in the treatment of endometriosis

**DOI:** 10.1530/RAF-21-0040

**Published:** 2021-09-23

**Authors:** Peter Thiel, Matthew J Burke, Philippa Bridge-Cook, Mathew Leonardi

**Affiliations:** 1 Department of Obstetrics and Gynecology, University of Saskatchewan College of Medicine, Regina, Saskatchewan, Canada; 2 Neuropsychiatry Program, Department of Psychiatry and Division of Neurology, Department of Medicine, Sunnybrook Health Sciences Centre, University of Toronto, Toronto, Ontario, Canada; 3 Division of Cognitive Neurology, Department of Neurology, Beth Israel Deaconess Medical Center, Harvard Medical School, Boston, Massachusetts, USA; 4 The Endometriosis Network Canada, Toronto, Ontario, Canada; 5 Department of Obstetrics and Gynecology, McMaster University, Hamilton, Ontario, Canada

**Keywords:** endometriosis, gynecology, nocebo effect, clinical effectiveness

## Abstract

The current approach to treating endometriosis is often inadequate or intolerable for many patients. Until more effective therapies are available, we should aim to maximize the effectiveness of our current options. Optimization may be possible by reducing nocebo effects, which are the negative therapeutic effects not directly caused by a treatment. Awareness of these effects, how they arise, and the factors influencing them, is invaluable if we aim to limit their magnitude. The unique nature of endometriosis diagnosis and management is especially prone to nocebo effects due to multiple factors, including diagnostic delays, feelings of invalidation, social transmission of expectations, and persistent symptoms despite numerous treatments. This commentary discusses the origins of these effects in people with endometriosis, methods of limiting nocebo effects, and future research directions.

**Lay summary:**

The term ‘nocebo’ describes the undesirable effects of a medication or treatment that patients may experience which are not directly caused by the treatment (e.g. tiredness from a sugar pill). These arise from pre-existing expectations toward a treatment and are influenced by multiple external factors, including past experiences, online media, personal beliefs, and personality factors. Endometriosis is a disease characterized by cells like those from the inside of the uterus growing outside of the uterus. The complex nature of endometriosis diagnosis and management creates an environment where nocebo effects may affect treatment outcomes. We may be able to limit nocebo effects through awareness and simple actions that strengthen patient–doctor relationships. Effective therapeutic relationships with doctors are crucial in limiting negative expectations and are established through empathy, honesty, and support. Therapeutic relationships built on trust may allow healthcare providers to address negative expectations, nocebo effects, and the misinformation affecting endometriosis management.

## Background

Nocebo effects are the undesirable effects or outcomes that patients experience due to treatment context rather than the treatment itself (Colloca & Barsky 2020). These are genuine symptoms that arise through physiological mechanisms involving the release of specific biologically active substances and alterations in brain activity and spinal cord signaling (Colloca & Barsky 2020). Nocebo effects are often non-specific and influenced by a myriad of personal and social factors, which leaves the treatment of endometriosis especially susceptible to their effects (Colloca & Barsky 2020).

Endometriosis is characterized by endometrium-like tissue outside the uterine cavity. It classically presents as cyclical pelvic pain, painful intercourse, and infertility; however, non-specific symptoms often accompany these more prominent features. Treatments in the domains of medical management, surgery, and alternative management can be effective for many if used optimally but may not achieve adequate symptom relief and produce intolerable side effects. Long-term efficacious treatments are even less common. Until we have more effective long-term, tolerable, and endometriosis-specific therapies available, we should strive to optimize our current therapeutic options. Knowledge of nocebo effects, how they are generated, and how to minimize them may serve this aim. In discussing these effects, we must be clear that we are not suggesting that patients fabricate reports of treatment ineffectiveness or the experience of side effects/complications. Not only is that not our intent but also it is simply not true. Patients’ experiences with treatments, whether effective or ineffective, are indeed authentic. With this in mind, our intention with this commentary is to bring awareness to nocebo effects in the treatment of endometriosis, recognize how our words and actions can affect treatment outcomes, discuss strategies to limit them, and suggest how to further our understanding of these phenomena in our field.

## The origin of nocebo effects

Patient-held negative expectations are the fundamental basis for nocebo effects. These expectations arise from a complex interplay of socio-psychological factors, including personal experiences, observational learning, personal beliefs, verbal suggestion, media, and personality factors. These individual factors form a patient’s unique mindset, shaping how they perceive, react to, and accept therapy options and suggestions (Petrie & Rief 2019). Online communities have become essential in supporting people with endometriosis and offer emotional support, connection, a source of information, and a place to share personal experiences (Shoebotham & Coulson 2016). Participation in online communities is a crucial component in the self-management of endometriosis with a net positive effect (Shoebotham & Coulson 2016). There are, however, concerns regarding the accuracy and credibility of information available online (Shoebotham & Coulson 2016). Unregulated spread of misinformation may lead to fear or negative expectations toward a specific treatment, influencing treatment effectiveness (Petrie & Rief 2019). Sharing personal experience with different treatments is also common in online communities and holds therapeutic benefits (Shoebotham & Coulson 2016). However, observation of unsuccessful treatment and interindividual spread of attitudes and beliefs toward treatment approaches may lead to nocebo responses (Petrie & Rief 2019). This social transmission of negative expectations toward specific therapies can increase the likelihood of experiencing those same effects (Petrie & Rief 2019).

The effect of negative expectations on the experience of side effects has been demonstrated in statin therapy, beta-blockers, and Aspirin (Wood *et al.* 2020). In fact, a recent trial of patients who had discontinued statin therapy because of side effects found that 90% of the side effects were attributable to nocebo effects (Wood *et al.* 2020). Consistent findings have been demonstrated in patients undergoing endocrine therapy (similar to some endometriosis treatments) following breast cancer surgery (Nestoriuc *et al.* 2016). Pre-treatment expectations of side effect intensity were positively associated with the severity of reported side effects, poor treatment adherence, and lower health-related quality of life (Nestoriuc *et al.* 2016). This study also found that pre-treatment side effect expectations were related to the experience of non-specific symptoms not directly attributable to the medication, a phenomenon known as symptom misattribution (Nestoriuc *et al.* 2016). All patients experience a wide variety of physical sensations daily, and the interpretation of these perceptions relies on contextual elements and expectations (Petrie & Rief 2019). Symptom misattribution results when these normal somatic sensations are perceived as negative side effects related to an intervention, medication, or other therapy (Petrie & Rief 2019). Misattribution is more prevalent in patients with higher degrees of symptom anxiety due to hypervigilant scanning for symptoms, thus contributing to a greater experience of side effects, both related and unrelated to the treatment (Petrie & Rief 2019).

Specific challenges associated with endometriosis diagnosis and management may also result in nocebo effects. For example, the delay in diagnosing endometriosis may result in many medical visits with limited benefit and feelings of invalidation. This lack of validation may lead to the expectation of not being heard and having symptoms downplayed. These negative attitudes can then spread to all aspects of the management plan and contribute to inadequate symptom relief, intolerable side effects, and treatment failure.

## Optimizing patient interaction

The first step in minimizing nocebo effects is awareness of their presence, and the core of our ability to influence their magnitude is the patient–practitioner relationship. Effective therapeutic relationships, which are crucial in limiting negative expectations, are established through empathy, validation, and honesty (Blasini *et al.* 2018, Petrie & Rief 2019). Non-verbal communication, including purposeful body language, limiting looking at a screen, active listening, and responsiveness, strengthens this therapeutic relationship (Blasini *et al.* 2018, Petrie & Rief 2019). Giving people with endometriosis time to tell their stories, hearing them, and emphasizing that they are being heard creates relationships where patients feel validated, supported, and respected. When discussing treatment options, positive framing should be used to review potential adverse outcomes (Petrie & Rief 2019). Positive framing is a technique where the proportion of patients who do not experience a side effect is communicated (e.g. 80% of patients do not experience mood changes; 95% of patients do not experience a serious surgical complication) (Petrie & Rief 2019). This technique effectively discloses potential side effects without inducing the negative expectations that lead to nocebo effects (Petrie & Rief 2019). Emphasizing the rapid reversibility of possible side effects may also contribute to successful medical therapy (Petrie & Rief 2019). Regardless of the treatment in consideration, a healthy therapeutic alliance is required to have open discussions that help identify attitudes toward treatment options and provide appropriately tailored care with a higher chance of success.

## Building trust

This patient–practitioner relationship relies on trust that is developed through working together to find acceptable and effective treatments. A breakdown in this trust can occur when patient’s and physician’s impressions of specific treatment strategies differ (Hirsch *et al.* 2017). When this difference in opinions arises, flatly dismissing a patient’s perspective is absolutely unacceptable. Dismissal leads to feelings of invalidation and distrust, which can negatively affect the patient–practitioner relationship and amplify nocebo effects (Blasini *et al.* 2018). Additionally, a trusting relationship with open communication has also been shown to palliate symptoms and reduce objective measures of inflammation (Colloca & Barsky 2020). Clinicians should seek to understand why a patient feels the way they do toward a treatment option. If we identify the source of a patient’s impression, we may better understand their expectations and beliefs toward specific treatment options. In the case of social media as the source, we should acknowledge the benefits of involvement in support groups and be aware of the potential for social transmission of side effect expectations.

## Addressing misinformation

The free access to information that the internet provides has dramatically reduced the role of clinicians in the dissemination of information. This access, coupled with the availability of inaccurate, outdated, and low-quality information accessible through internet searches, creates an environment ripe for negative expectation generation (Hirsch *et al.* 2017). The regulation of this information poses a significant challenge, as evidenced by attempts to curtail its spread during the COVID-19 pandemic (Tangcharoensathien *et al.* 2020). Rather than try and regulate the information that patients have access to, we should collaborate with the prominent social media pages and websites that patients are accessing to ensure that they are up-to-date and factual (Tangcharoensathien *et al.* 2020). This collaboration with endometriosis communities could also aid in identifying areas of concern that would benefit from tailored messaging (Tangcharoensathien *et al.* 2020). Furthermore, the visibility of healthcare providers working hand-in-hand with advocacy groups in the online sphere may demonstrate to people with endometriosis that healthcare providers equally respect this space, value its contribution to education, and want to actively participate for the greater good. However, patients also need access to support systems that are free from medical influence, and this collaboration may be seen as an intrusion and could negatively affect trust in the medical field.

We believe that the best way to address misinformation is through a trusting patient–practitioner relationship; this allows the clinician’s message to be optimally heard by the patient. No amount of high-quality evidence from an *untrusted* physician will sway a patient from the ideas and beliefs developed through interactions with other trusted sources.

## Conclusions and future directions

Nocebo effects result from negative expectations arising from a person’s unique set of lived experiences influenced by personal and social factors. We currently have a poor understanding of how prevalent and significant nocebo effects are in treating endometriosis. More research is needed to determine what factors influence the development of negative expectations in this patient population. Specifically, the impact of the internet and social media on expectations will become increasingly relevant as online communities continue to grow. Equally important will be furthering our understanding of the effect of patient’s expectations on the medical and surgical treatment of endometriosis. While we actively contribute to answering these questions scientifically, it seems reasonable to strive for therapeutic relationships that foster effective patient-centered care, which may limit nocebo effects. That process starts with awareness, building strong therapeutic alliances, listening to our patients, dispelling misinformation, and working with them to find effective and acceptable treatments. With these simple concepts in mind, we can provide individualized care and further our ability to effectively treat and manage people with endometriosis.

## Declaration of interest

M L reports receiving a research support grant from the Australian Women and Children’s Research Foundation and consulting fees from GE Healthcare, unrelated to this work. Mathew Leonardi is an Associate Editor of Reproduction and Fertility. Mathew Leonardi was not involved in the review or editorial process for this paper, on which he is listed as an author. The remaining authors have no conflicts of interest to report.

## Funding

This work did not receive any specific grant from any funding agency in the public, commercial, or not-for-profit sector.

## Author contribution statement

P T and M L conceived and planned the commentary. P T drafted the article with M B, P B, and M L all contributing revisions and significant intellectual content. All authors provided approval of the final version for publication.
